# Absence of a bilingual cognitive flexibility advantage: A replication study in preschoolers

**DOI:** 10.1371/journal.pone.0255157

**Published:** 2021-08-05

**Authors:** Anahita Shokrkon, Elena Nicoladis

**Affiliations:** Department of Psychology, University of Alberta, Edmonton, AB, Canada; Nagoya University, JAPAN

## Abstract

Some studies have found a bilingual advantage in children’s executive function and some failed to find a bilingual advantage. For example, the results of a previous study by Bialystok & Martin (2004) indicated that Chinese-English bilingual preschool children outperformed English monolingual children in solving the dimensional change card sort (DCCS). The goal of our study was to replicate this study using the same dimensional change card sort task. We also tested our participants on vocabulary and digit span. Our participants were 40 English monolingual and 40 Mandarin-English bilingual children and were within the same age range as the children in Bialystok & Martin’s (2004) study. Our results showed no difference between bilinguals and monolinguals. Both groups of children in the present study performed better than those in Bialystok and Martin (2004), but the bigger difference was between the two groups of monolinguals. These results suggest that it could be important to attend to monolingual children’s performance, in addition to bilinguals’, when testing for a bilingual advantage. Our replication study is important because it helps with clarifying the validity of studies finding a bilingual advantage and to help future researchers know whether to build on their findings or not.

## Introduction

Many studies have found that bilinguals have enhanced cognitive abilities relative to monolinguals [1–6]. Studies have found bilingual advantages on spatial problems [7], mental flexibility [8], metacognitive skills [9], learning strategies [10], and executive function (EF). EF is a set of cognitive processes that regulate one’s thoughts and enable engagement in goal-directed behaviors [11, 12]. Although there are many conceptualizations of EF components, there is general agreement that EF consists of three main components, attention and inhibition (the ability to control attention, behavior, and thoughts), working memory (a mental workplace in which information is held temporarily and mentally manipulated) and cognitive flexibility (allows us to think divergently, change perspective and adapt to a constantly changing environment) [13]. Bilingual advantages have been found in all three aspects of EF: inhibition [5, 14–18], shifting [6, 19–21], and working memory [16, 22, 23]. The bilingual advantage has been attributed to bilinguals’ greater practice in inhibitory processes and attentional control in processing their language(s), such as inhibiting the non-target language [24] or actively attending to the target language [25].

However, many studies have failed to find a bilingual advantage on cognitive measures [26–30]. The lack of bilingual advantage has also been found in all three domains of EF: inhibition [28, 31–33] shifting [28, 31], and working memory [34–36]. Researchers have proposed a number of explanations for these discrepant findings, including physical activity and dietary intake [37, 38], culture [39, 40], the age of participants [27], demographic factors [29, 33, 41], weak psychometric properties of cognitive tasks [28, 41], sample size [42], and publication bias [43].

Since there are somewhat contradictory results in the field of bilingualism and EF, to explore whether speaking more than one language actually impacts the development of EF, one enlightening step is replication [44–47]. As the recent *replication crisis* gave rise to the importance of replication in order to evaluate whether published results reflect true findings or false positives, the purpose of the present study is to replicate a highly cited study by Bialystok and Martin (2004) showing a bilingual advantage [19] with a very similar population from the same country and using the same measures. This approach is also justified in light of the limited reproducibility of research in the field of psychology [48].

In their study, Bialystok and Martin (2004) compared 36 English monolinguals (18 boys and 18 girls, M = 59.1 months) and 31 Chinese-English bilinguals (21 boys and 10 girls, M = 58.9 months) [19]. They measured the children’s receptive vocabulary in English (with the Peabody Picture Vocabulary Test) and verified that the children did not differ on verbal working memory capacity (with a forward digit span task). To assess the children’s attention and inhibition component of EF, they administered the dimensional change card sort (DCCS) task. The DCCS is a commonly used measure of EF in preschool children [48]. In this task, children are asked to sort cards with pictures (such as red and blue rabbits and boats) according to one dimension of the pictures (such as color) and, then asked to sort the cards according to another dimension (such as shape). This task requires children to attend selectively to the relevant features of a problem, inhibit attention to irrelevant information, and switch between rules. When asked to switch rules, young children typically perseverate, meaning that they continue to apply the original rule even after the rules have switched [48]. The results of Bialystok and Martin (2004) showed that the bilinguals performed better than monolinguals at switching rules. In other words, they found a bilingual advantage [19].

### The present study

The main objective of the present study is to replicate previous work by Bialystok and Martin (2004), using the same tasks and with a very similar population. In this study, we compared Mandarin Chinese-English bilingual children’s performance on the DCCS to that of English monolingual children. The children were within the same age range as the children in Bialystok and Martin (2004) [19]. We expected to replicate their results and find a bilingual advantage in attention and inhibition component of EF.

## Materials and methods

### Participants

80 children were included in the analyses: 40 Mandarin Chinese-English bilingual children (16 girls and 24 boys) and 40 English monolingual children (23 girls and 17 boys), picking the closest matching on age to the bilinguals from a database of 79 English monolinguals. Both the monolinguals and the bilinguals were living in Edmonton, Alberta, Canada. The bilinguals averaged 63.5 months of age (SD = 9.4; range: 48–80) and the monolinguals averaged 62.0 month (SD = 7.4, range: 47–83). The bilingual children were sequential bilinguals, having first learned Mandarin at home with their families. They started to learn English between the ages of two and four years, usually when they started daycare or preschool.

In spite of the fact that the data was originally collected for another study and we did not have a direct measure of socioeconomic status (SES) of the children’s families, we had attempted to control for SES through our recruitment methods. The monolingual participants were recruited from daycares close to the university that are known to have many children of academics. The bilingual participants were all children of graduate students and postdoctoral fellows. Thus, most of our participants were likely from high SES families. We discuss possible effects of SES in the Discussion.

### Procedure

This study was reviewed for its adherence to ethical guidelines by a Research Ethics Board at the University of Alberta (Pro00040247). Parents gave us permission to test their children by signing consent forms. Also, verbal assent was asked from the children before testing. The order of the tests within a testing session depended on each child and the experimenter, but we started with more passive tasks, such as the receptive vocabulary test and then continued with the Dimensional Change Card Sort task.

### Measures

#### Vocabulary

The Peabody Picture Vocabulary Test III (PPVT) was used to measure receptive vocabulary size [49]. In the present study, we report the children’s raw scores.

#### Working memory

The Wechsler Intelligence Scale for Children (WISC-R) forward digit span subtest was used to measure verbal working memory [50]. In this test, a random string of digits was presented to children and they were asked to repeat the exact strings of digits. The test started with one digit. If the child repeated back the digit correctly then another digit was added to the string to be remembered. After one error, the test ended. The score represents the highest number of digits the child repeated without errors. The possible range of scores for this measure is 0–8; the actual range of scores was 2–7.

#### Executive function measure

We used the same Dimensional Change Card Sort (DCCS) as Bialystok and Martin (2004) for children in the same age range as the present study [19]. For this task, children were asked to sort a set of 10 cards into two groups based on one characteristic of the stimuli and then to resort the same set of 10 cards based on a different characteristic. The task was comprised of the following four conditions or games:

1. *Colour game*. In this condition, the stimuli were five red squares and five blue squares. The children were first told to sort the cards according to their colour. So, the experimenter told them: “This is the color game. In this game, the red ones go here and the blue ones go here” After 10 trials, children were told that they were going to play a new game with new rules. They were told: “In this game, the red ones go here (in the place they used to put blue cards) and the blue ones go there (in the place they used to put red cards).”
2. *Color-shape game*. The stimuli were blue squares and red circles. In the pre-switch phase, there were two boxes with a target stimulus on them. One box had a picture of a red square and the other box had a picture of a blue square. In the pre-switch game, the children were told to place all the blue cards in the box with the picture of the blue square and all the red cards in the box with the picture of the red square. In the post-switch phase, children were told to put all the square cards in the box with the picture of a square and all the circle cards in the box with the picture of a circle.
3. *Color-object game*. This game was the same as the previous game except that meaningful objects were used. The stimuli were red flowers and blue rabbits and the two boxes had the pictures of a red rabbit and a blue flower.
4. *Function-location game*. In this game, instead of perceptual characteristics, the sorting dimensions were abstract features of the stimuli (function vs. location). The set included five things to play with that went outside the house (bicycle, skateboard, pail and shovel, skipping rope, kite) and five things to wear that went inside the house (slippers, nightgown, bib, ballet shoes, and baby pyjamas). On the sorting boxes, there were pictures of a teddy bear (play-inside) and a winter jacket (wear-outside). In the location game, the children were told to put the things that go inside the house in the box with the picture of the teddy bear and the thing that goes outside the house in the box with the picture of the jacket. In the function game, children were told to put the thing to play with inside the box with the picture of the teddy bear and the things to wear in the box with the picture of the jacket.

The first two conditions of this DCCS are similar to the standard DCCS described by Zelazo (2006) [48]. By the age of five years, almost all children can switch rules easily for the colour and shape games [48, 51]. Indeed, one study found that 92% of four-year olds could successfully switch rules for a DCCS similar to the first two conditions [52]. As far as we know, the latter two conditions are unique to Bialystok and Martin (2004) [19]. There was one difference between the DCCS task we used and that of Bialystok and Martin (2004): we administered it on paper and they administered it on a computer. Bialystok and Martin (2004) asserted that the computer administration made the task age-appropriate [19]. We keep in mind that possibility as we interpret the results.

## Results

The bilinguals scored significantly lower (M = 33.5, SD = 19.6) than the monolinguals (M = 89.3, SD = 26.4) on the PPVT, t(77) = -10.53, *p* < .001. The bilinguals did not differ on the forward digit span (M = 4.5, SD = 1.0) from the monolinguals, t (M = 4.4, SD = 0.8), *t*(78) = .73, *p* = .47).

**Fig 1:**
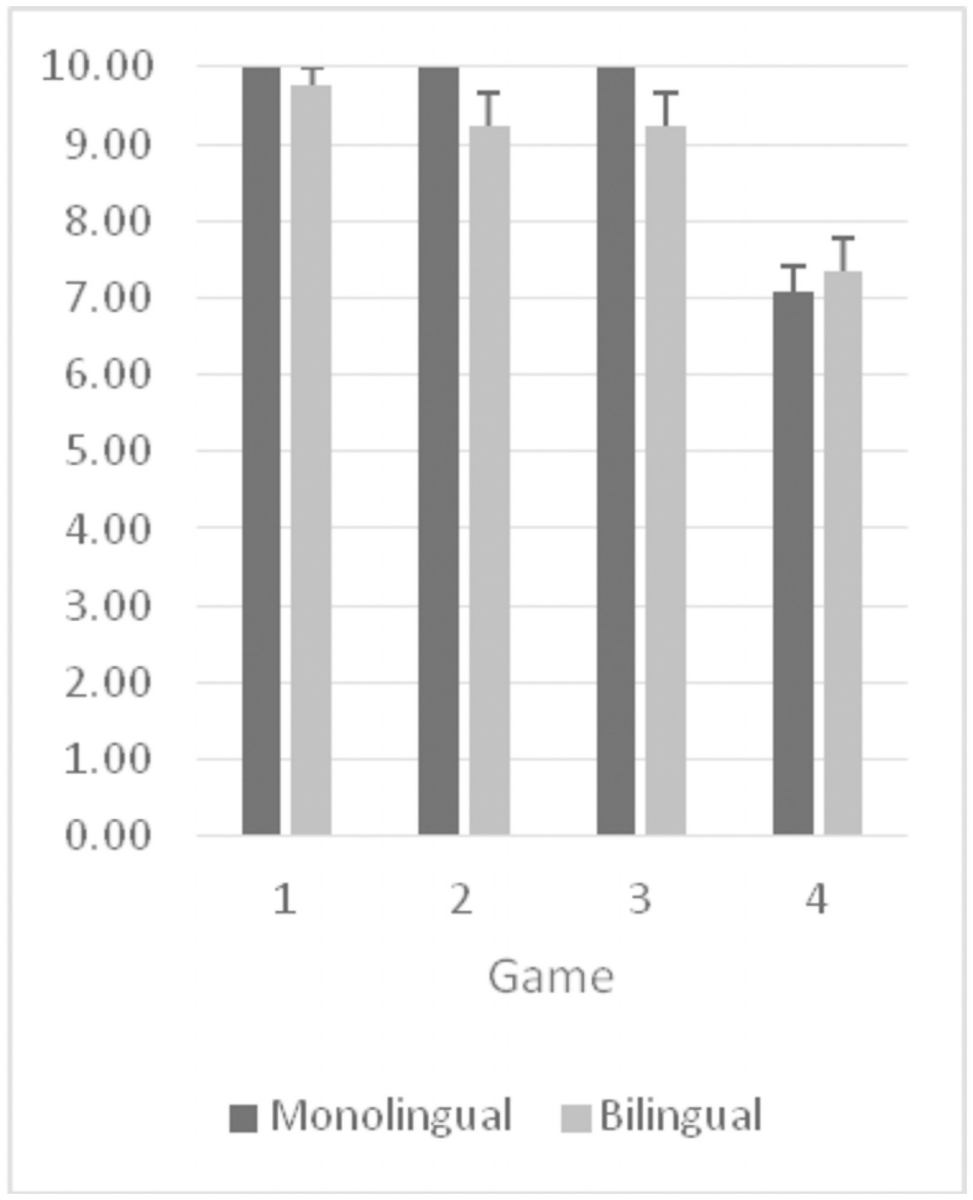
DDCS pre-switch scores at the four levels of difficulty for bilinguals and monolinguals.

Then we examined the scores in a three-way ANOVA for game (4), pre/post (2) and language group (2), with the first two variables as repeated measures, as Bialystok and Martin (2004) did [19]. The results showed only a main effect of game, *F* (3, 117) = 60.05, *p* < .001. Post-hoc contrast analyses showed that the fourth game (the function-location game) was the most difficult game, while the other three games were not different from each other. The main effect results of pre/post, *F* (1, 39) = 3.83, *p* = .14, and language groups, *F* (1, 39) = 2.40, *p* = .12. The only interaction to reach significance was between game and phase, *F* (3, 117) = 2.74, *p* = .03. This interaction is likely due to the children’s worse performance on the fourth game following the rule change than before the rule change, while there was little change for the other four games (see Fig 1).

**Fig 2:**
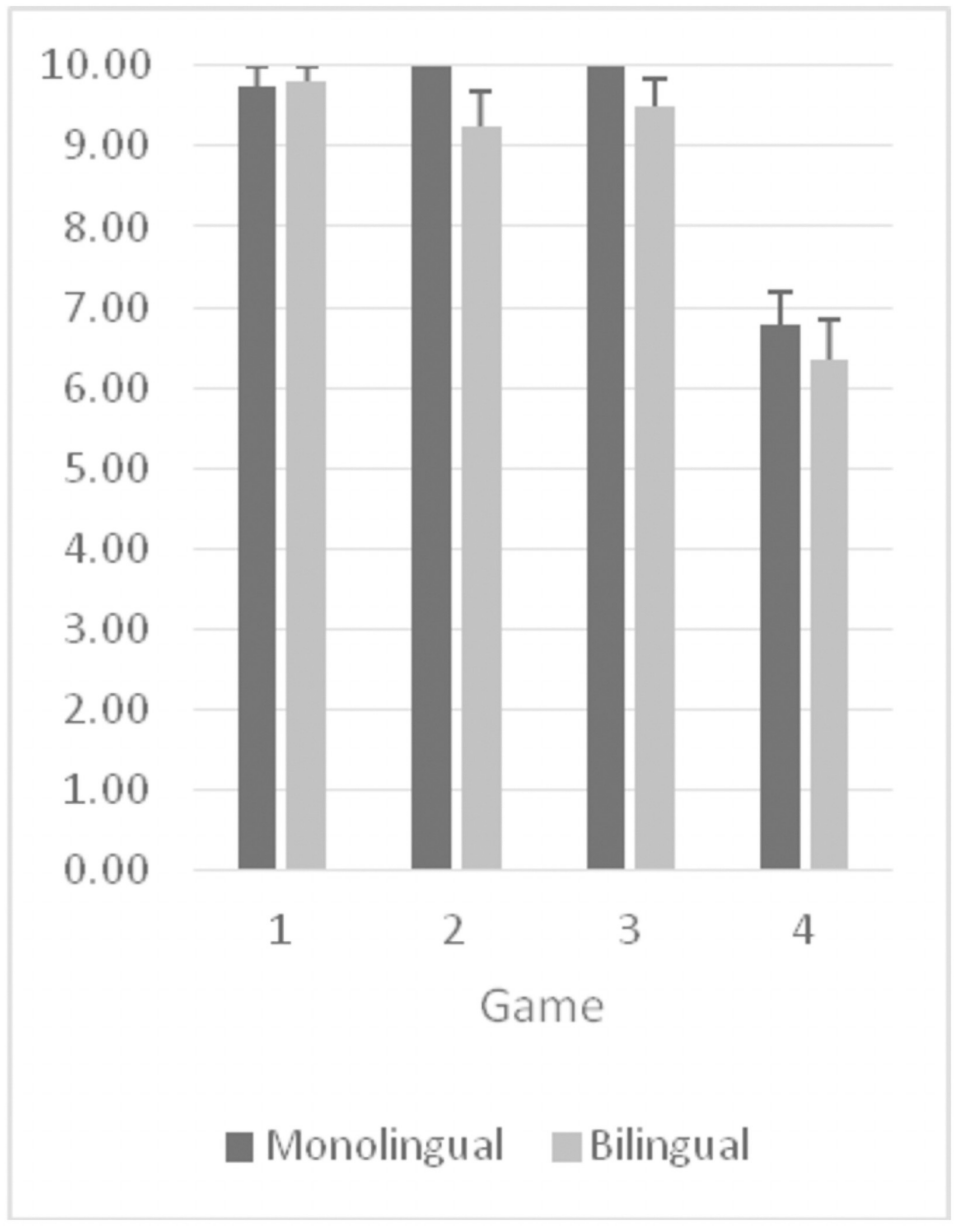
DDCS post-switch scores at the four levels of difficulty for bilinguals and monolinguals.

Figs 1 and 2 summarize the children’s number correct (out of 10) at each level of difficulty both before switching rules (Fig 1) and after switching rules (Fig 2).

As Bialystok and Martin (2004) did [19], we next divided the children in our study into perseverators (0–3 on post trials), guessers (4–6) and correct (7–10). We then performed chi-squares comparing the distribution between bilinguals and monolinguals (see Table 1). There were no significant differences between groups.

**Table 1:** Monolinguals and bilinguals performance in DCCS task and Chi-square results.

Condition	Language group	Perseverators	Guessers	Correct	Chi-square†
Colour	Monolingual	1	0	39	0.00, *ns*
	Bilingual	1	0	39	
Colour-shape	Monolingual	0	0	40	1.01, *p* = .29
	Bilingual	3	0	37	
Colour-object	Monolingual	0	0	40	0.37, *p* = .54
	Bilingual	2	0	38	
Function-location	Monolingual	5	10	25	1.39, *p* = .50
	Bilingual	9	9	22	

^†^df = 1 for all but the function-location game where df = 2

We next compared the distribution in our study with that of Bialystok and Martin’s (2004) [19]. The results for the bilinguals are summarized in Table 2, for the monolinguals in Table 3. As can be seen in Table 2, our bilinguals scored higher than theirs for the first three levels of the DCCS, but not the fourth. However, as can be seen in Table 3, our monolinguals scored much higher than the monolinguals in Bialystok and Martin (2004), significantly higher on all four levels of the DCCS [19].

**Table 2:** Distribution comparison of the bilinguals in the present study and Bialystok and Martin’s (2004) study.

Condition	Study	Perseverators	Guessers	Correct	Chi-square (df = 2)	Chi-square (df = 1)†
Colour	Our study	1	0	39	12.02, *p* = .003	6.70, *p* = .01
	B&M	6	4	21		
Colour-shape	Our study	3	0	37	7.67, *p* = .02	5.06, *p* = .02
	B&M	8	2	21		
Colour-object	Our study	2	0	38	14.38, *p* = .0008	13.13, *p* = .0003
	B&M	12	1	18		
Function-location	Our study	9	9	22	1.31, *p* = .52	0.56, *p* = .45
	B&M	8	10	13		

^†^Comparing only the perseverators and correct

**Table 3:** Distribution comparison of the monolinguals in the present study and Bialystok and Martin’s (2004) study.

Condition	Study	Perseverators	Guessers	Correct	Chi-square (df = 2)	Chi-square (df = 1)†
Colour	Our study	1	0	39	25.79, *p* < .001	2.75, *p* = .10
	B&M	4	4	28		
Colour-shape	Our study	0	0	40	35.60, *p* < .001	23.33, *p* < .001
	B&M	14	6	16		
Colour-object	Our study	0	0	40	37.84, *p* < .001	34.50, *p* < .001
	B&M	21	2	13		
Function-location	Our study	5	10	25	29.25, *p* < .001	9.63, *p* = .002
	B&M	17	5	14		

^†^Comparing only the perseverators and correct

Finally, Table 4 summarizes the correlations between post-switch game 4 and age, vocabulary, and forward digit span. For the other three games, there was not enough variability to perform correlations. As can be seen in Table 4, forward digit span is the only significant predictor for both bilinguals and monolinguals. In addition, age and vocabulary are significantly correlated with only bilinguals’ performance.

**Table 4:** Correlations between post-switch game 4 and age, vocabulary, and forward digit span.

	Monolinguals	Bilinguals
Age	.23	.55**
Vocabulary	.24	.33*
Forward digit span	.39*	.46**

* p < .05;

** p < .01

## Discussion

The purpose of the present study was to replicate Bialystok and Martin (2004). As the inconsistencies around the hypothesis of “bilingual advantage” have risen in recent years, it is important to replicate the results of the existing studies to make sure about the validity of studies and to help future researchers know whether to build on their findings or not.

Our participants were within the same age range as Bialystok and Martin (2004), averaging just a few months older and we had a slightly larger sample size. Our participants were from the same country as Bialystok and Martin (2004) and we used the same dimensional change card sort (DCCS) task as they did, with the only difference that we used the paper version of the test rather than the computer version.

Our results showed no bilingual advantage on the DCCS in this study (as have other studies; 28, 31–33]. Furthermore, on the DCCS task, while our bilinguals were slightly better than the bilinguals in Bialystok and Martin (2004), our monolinguals performed much better than theirs. It is possible that the computerized version of the DCCS was harder for the children (as claimed by Bialystok & Martin, 2004). If so, the greater difficulty might account for the difference between the two bilingual groups. However, the difference between the two monolingual groups was much larger, suggesting that there was some other factor(s) at play. Moreover, the monolinguals in our study performed at ceiling on the first levels of the DCCS, as has been reported in similar studies with monolinguals of this age [49, 51].

We speculate that there could be multiple factors responsible for our monolinguals doing better than their monolinguals. First, Paap (2014) showed that most studies showing a bilingual advantage were small-scale studies (average n = 29) [53]. The small number of participants may reduce statistical power, and false-positive findings are more commonly found in small sample studies [54, 55]. Many studies with large sample sizes have not shown a bilingual advantage [32, 56, 57]. Neither our study nor that of Bialystok and Martin (2004) could be characterized as large, so a future study with a larger sample size could decide which results are more replicable. Second, not all studies showing a bilingual advantage have used measures with strong psychometric properties. For instance, many of these studies have used measures with low levels of convergent and discriminant validity [42]. For instance, using the Flanker task, Poarch and Van hell found that bilinguals performed better than monolingual in inhibitory control [58]. Later, they reanalyzed their data and figured that congruent and incongruent conditions and the difference score across tasks were not correlated, which in turn raises doubt about the convergent validity across tasks [59]. Paap, Anders-Jefferson, Zimiga, Mason, and Mikulinsky (2020) also posited that Flanker, Simon, and spatial Stroop tasks should not be characterized as reflecting inhibitory control [60].

Also, studies testing EF can be compromised by weak test-retest reliability [41]. The DCCS (the first two games in the present study) has been shown to have high test-retest reliability [48], however only for three-year-olds. In the present study, it was not entirely clear that DCCS was tapping EF at all. While the children did show some small declines on the post-test relative to the pre-test, only 14 out of the 80 children (18%) were classified as perseverators. The DCCS is thought to tap EF by showing that children continue to follow the same rule as before the rule change [48]. Children’s post-change performance on the fourth game was strongly correlated with their digit span, suggesting that the DCCS administered here might have been a measure of short-term memory. Finally, another possible reason for our contradicting results with those of Bialystok and Martin (2004) might be related to SES of our participants. Bialystok and Martin (2004) claimed that their monolingual and bilingual participants were from similar SES backgrounds because they lived geographically close to each other [19]. No further information was provided about the participants; therefore, it is not known if the participants lived in well-off or poor neighborhoods. Previous research has indicated that SES is associated with EF [33, 61, 62]. In the present study, we tried to control for SES across the two language groups by recruiting participants who were connected to the university. It is possible that our study included monolinguals from higher SES backgrounds than those in Bialystok and Martin (2004) [19]. Future studies can include individual measures of the families’ SES to test this possibility.

This study has some limitations that need to be considered. First, SES was only semi-controlled as we did not have a direct measure of SES, and instead we attempted to control for SES through our recruitment method. Second, the number of our participants were not much higher than Bialystok and Martin (2004), therefore, a future study with a larger sample size could decide which results are more replicable. Another limitation of our study is that we cannot completely dismiss the possibility of a ceiling effect resulting from our participants being 3 months older than those in Bialystok and Martin (2004) and being from a higher SES family.

Regardless of these limitations, the current study attempts to address the question of whether speaking more than one language actually impacts the development of EF and makes important contributions to the literature suggesting that there might be mediating variables that explain our failure to replicate.

## Conclusion

The present study did not replicate the bilingual advantage on executive function performance found in Bialystok and Martin (2004), as both the bilingual and monolingual children in the present study outperformed those in Bialystok and Martin (2004) [19]. However, the monolingual children in the present study performed much better than those in Bialystok and Martin (2004) and closer to the performance of monolinguals from other studies [19]. Thus, it is important to verify that monolingual children perform at an age-appropriate level before concluding that there is a bilingual advantage. Moreover, future researchers in the field of bilingualism and EF, should pay more attention to using age-appropriate and real-life tasks measuring clearly reflected EF components. Also, more attention should be paid to the process of experimental set-up, design, procedure, data collection (e.g., trimming; how outliers are identified and excluded from further analysis), and the choice of statistical analyses which could lead to more variability in research findings [46].
